# Anthropometric and Health-Related Behavioral Factors in the Explanation of Social Inequalities in Low Birth Weight in Children with Prenatal Alcohol Exposure

**DOI:** 10.3390/ijerph110100849

**Published:** 2014-01-08

**Authors:** Manuela Pfinder

**Affiliations:** 1 Bielefeld Graduate School in History and Sociology, Faculty of Sociology, University of Bielefeld, P.O. Box 10-01-31, Bielefeld 33501, Germany; E-Mail: manuela.pfinder@uni-bielefeld.de; Tel.: +49-176-6155-1512; 2 Department of Pediatrics, University Hospital Munster, Albert-Schweitzer-Campus 1, Munster 48129, Germany

**Keywords:** low birth weight, social inequalities, alcohol, pregnancy, smoking, maternal height

## Abstract

There is evidence for social inequalities in the health status of children with prenatal alcohol exposure (PAE). This study aimed to describe social inequalities in low birth weight (LBW) in children/adolescents with PAE and to examine the contribution of anthropometric and health-related behavioral factors to the explanation of social inequalities. A total of 2,159 participants with parental self-reported moderate to regular PAE (enrolled in the cross-sectional German Health Interview and Examination Survey for Children and Adolescents) were examined. At similar levels of PAE, the risk of LBW was significantly increased in subjects with a low socioeconomic status (SES) (adjusted odds ratio (OR) 2.78, 95% confidence interval (CI) 1.59, 4.86) and middle SES (adjusted OR 2.04, 95% CI 1.28, 3.24). Maternal height, maternal body mass index (BMI) and smoking during pregnancy mediated the association. The mediating effect of maternal height was 12.5% to 33.7%. Maternal BMI explained 7.9% of the socioeconomic difference in LBW between the high and low SES groups in children with PAE. The mediating effect of smoking during pregnancy was 17.3% to 31.5%. Maternal height, maternal BMI and smoking during pregnancy together explained 24.4% to 60.1% of the socioeconomic differences in LBW in children with PAE. A large proportion of the socioeconomic differences in LBW in children with PAE can be attributed to anthropometric and health-related behavioral factors.

## 1. Introduction

It is known that maternal alcohol intake during pregnancy is associated with a broad range of irreversible disorders and serious impairments in the offspring since Lemoine *et al*. [1] reported on the adverse effects of prenatal alcohol exposure (PAE); these effects are covered by the umbrella term “fetal alcohol spectrum disorders” (FASD). Growth retardation and low birth weight (LBW) were among the first observed negative effects of PAE [1,2,3,4,5,6,7,8,9,10]. Although alcohol intake during pregnancy was reported to be most prevalent in women with a high socioeconomic status (SES) [11,12,13,14,15,16,17], the adverse effects of PAE appeared mostly in the offspring with a low socioeconomic background [18,19,20,21,22,23,24,25]. A study on the influence of socioeconomic factors on the effects of PAE showed that the risk of giving birth to a child with alcohol-related disorders, alcohol-related birth defects and fetal alcohol syndrome (FAS), at similar levels of maternal alcohol intake during pregnancy, is up to 15.8 times higher in women from the lower social class compared with women with a high SES [20]. For instance, mean birth weight was shown to be significantly decreased in alcohol exposed subjects from the lower social class compared with those from the upper social class [20]. In turn, LBW was found to be associated with a considerable number of diseases over the life course, as described by the theory on fetal origins of adult diseases (FOAD) [26,27,28,29,30,31,32,33,34]. In line with the FOAD theory, a study from Germany reported that the risk of alcohol-related disorders in children with similar levels of PAE is 2.4 times higher in those with LBW [34]. Thus, LBW may account for various adverse outcomes associated with FASD over the life course of individuals with PAE. This emphasizes the need to explore why individuals exposed to comparable levels of alcohol in utero show a higher risk of LBW when they have a lower socioeconomic background.

Although SES has no direct effect on LBW, a lower SES can be seen as a proxy for risk factors that cause LBW, such as adverse conditions and health-related behaviors. Reasons for the increased risk of LBW in offspring with PAE might be maternal health-related behavioral factors and parental anthropometrics. Both types of factors are hypothesized to contribute to the explanation of social inequalities in LBW in children with PAE. In addition, smoking during pregnancy is a known adverse health-related behavior. The association between the risk of LBW and maternal smoking during pregnancy is well established [35,36,37,38,39,40,41], suggesting an increased risk of LBW in the offspring of smokers. Smoking during pregnancy is also strongly associated with SES, indicating a higher prevalence of smokers during pregnancy in the lower social class [35,42]. Therefore, it is reasonable to hypothesize that tobacco smoke exposure during pregnancy mediates the socioeconomic differences in LBW. As smoking during pregnancy was found to exacerbate the effect of PAE on LBW [18,19,43,44,45,46], its contributory power to the socioeconomic differences in LBW might be stronger in subjects with PAE than in the more general population. Therefore, it is necessary to test this hypothesized mediation in a subsample of subjects with PAE.

Besides maternal smoking during pregnancy, parental anthropometrics may also play a role in the association between SES and LBW. Parental anthropometrics [height, weight, body mass index (BMI), gestational gain in weight] are known to affect the offspring’s birth weight [39,41,47,48,49,50,51,52,53]. The association between increasing gestational gain in weight and increasing birth weight is also well established [41,43,45]. Shorter women and underweight women are at higher risk of giving birth to a child with LBW compared with their “average” counterparts [39,50,54]. Paternal height and weight are also relevant predictors of birth weight, suggesting that offspring’s birth weight increases with increasing height and weight of the father [50,52]. Unfavorable anthropometric conditions, such as insufficient gestational gain in weight, short stature and very low weight (a BMI below the recommended levels), are more prevalent in the lower social class [55,56,57]. The SES of an individual is highly correlated over the life course and predicts anthropometric conditions which remain fixed (to some extent) at a certain point in life (e.g., height) and these factors may, in turn, account for the risk of LBW. Therefore, it is reasonable to hypothesize that parental anthropometric factors mediate the socioeconomic differences in LBW. As maternal height and weight/BMI have an impact on the levels of blood alcohol concentration (BAC) and these factors alter the levels of BAC in the fetus [24], the contributory power to the socioeconomic differences in LBW might be stronger in individuals with PAE than in the more general population; therefore, it is necessary to test the hypothesized mediation in a subsample of subjects with PAE.

All anthropometric and health-related behavioral factors may account for social inequalities in the health of children with PAE, if they are related to LBW and socioeconomic conditions. 

To examine the question as to why the risk of LBW is greater in offspring with a lower SES at similar levels of PAE, it is necessary to focus on subjects with PAE only, as a multiplicity of risk factors associated with a low SES increase a person’s vulnerability to the adverse effects of PAE by creating a biological milieu within which alcohol develops its major effects [18,19,22,23,24,25]. 

An explanation of the socioeconomic variations in LBW in children with PAE is essential to understand and make recommendations for public health actions to reduce social inequalities in LBW among children with PAE, and to avoid an increase of health inequalities over the life course. Although social inequalities in LBW among alcohol-exposed newborns have been reported, the present study is the first to investigate an explanation for the socioeconomic differences in LBW.

The aim of this study was to detect whether social differences exist in the distribution of LBW among children with PAE, and to explain social inequalities in LBW by identifying the contributions of health-related behavior and parental anthropometrics.

## 2. Methods

### 2.1. KIGGS Study

This study is based on data from the German Health Interview and Examination Survey for Children and Adolescents (KiGGS); this is a nationally representative cross-sectional study including 17,641 children/adolescents (aged 0–17 years) and their parents. The KiGGS study was commissioned by the German Federal Ministry of Health and was designed and conducted by the Robert Koch-Institute. The design of the survey, the process of recruitment and the fieldwork are described in detail elsewhere [56]. Briefly, four study teams followed a random route plan of 167 primary sample points in the years 2003–2006. The study consists of the KiGGS core survey and five additional in-depth modules with subsamples of the main study. Age-appropriate questionnaires were designed to be filled in by the parents, or by the children/adolescents when aged ≥11 years. Additional information on the health status of the children and adolescents was gained from medical examinations, anthropometric measurements and laboratory tests. Of the total sample of 28,299 participants, 17,641 children/adolescents and their parents were surveyed (response rate 66.6%).

Written informed consent according to the Helsinki Declaration was obtained from the participants and their parents or guardians before the individuals entered the study. Approval for the KiGGS study was obtained from the Ethics Committee of the Charité/Universitätsmedizin Berlin (Germany) and the Federal Office for the Protection of Data (20 February 2003).

### 2.2. Sample

Of the 17,641 participants, 2,331 individuals were exposed to alcohol before birth. PAE was measured through retrospective parental self-reports, questioning whether the mother drank alcohol during pregnancy. Possible answer categories were: “regular”, “moderate” or “no alcohol” intake during pregnancy. The terms “moderate” and “regular” are derived from self-report and may, therefore, be affected by the respondent’s subjective evaluation of such quantities. Participants without information on SES or birth weight, or information provided by non-biological parents and twin and multiple births were excluded from the analysis. This left a study population of 2,159 subjects with PAE for the analysis. 

### 2.3. Variable Measurements

#### 2.3.1. Low Birth Weight (LBW)

Parents were asked to give information on the birth weight of their child. LBW was set to ≤ 2,700 g after calculating the 10th percentile based on information from 16,877 questionnaire responders in the overall cohort study on birth weight [34]. Similar to other studies, LBW was defined by the 10th percentile of birth weight in the entire study population [35,36]; the result was comparable to the 10th percentiles of birth weight in a study from Denmark (≤2,700 g and ≤2,910 g, respectively) [35] and from Italy (<2,600 g) [57]. 

#### 2.3.2. Socioeconomic Status (SES)

The SES was measured with Winkler’s index [58,59] which was readjusted for the KiGGS study [60]. Winkler’s index is a widely used social class index based on the validated Scheuch index [61]. Winkler’s index is defined and measured by the net income, the basic and vocational education, and by the profession. Each of the three dimensions scores 1–7 points. The index ranges from 3–21 points: 3–8 points indicate the low SES group, 9–14 points indicate the middle SES group, and 15–21 points indicate the high SES group [61,62]. The variable is derived from the main wage earner in the household and is categorized into high, middle and low. Further details on the measurement and classification can be found in [62].

#### 2.3.3. Anthropometrics

Parental anthropometrics might be associated with the birth weight of the child and the SES. Gestational gain in weight (categorical variable: low (<10 kg), medium (10–15.9 kg), high (>16 kg)) [54], maternal height (continuous variable), paternal height (continuous variable), maternal BMI (categorical variable: underweight (BMI < 18.5), not underweight (BMI ≥ 18.5)) [63] and paternal BMI (categorical variable: underweight (BMI < 18.5), not underweight (BMI ≥ 18.5)) [63] were measured by parental self-report questionnaires.

#### 2.3.4. Maternal Health-Related Behaviors

Information on maternal health-related behaviors during pregnancy was restricted to maternal smoking during pregnancy (categorical variable: no, moderately, regularly) and was measured by self-report questionnaires.

#### 2.3.5. Covariates

The models were adjusted for maternal age at child’s birth (continuous variable), gender of the child (categorical variable: male, female), ethnicity (categorical variable: German, Slavic, Turkish, Others) and parity (categorical variable: 0, >0). 

### 2.4. Statistical Analyses

Potential mediation was tested by the Baron and Kenny criteria [64]. Each of the anthropometric and health-related behavioral factors is accepted as a mediator: (1) if the variable is affected by the independent variable; (2) if the independent variable affects the dependent variable; (3) if the mediator affects the dependent variable; and (4) if the effect of the independent variable on the dependent variable is decreased after adjustment for the mediator.

In the first stage, according to Baron and Kenny [64], the frequency distribution by levels of SES (independent variable) of anthropometric and health-related behavioral factors (potential mediators) and confounders was established (first criterion). Also, the association between the SES and LBW (dependent variable) was tested (second criterion). The one-way-analysis of variance (ANOVA) was applied to test trends across levels of SES for continuous factors and the chi-squared test was used for categorical factors (Table 1). The univariate association between anthropometric and health-related behavioral factors and LBW was examined by applying the ANOVA for continuous factors and the chi-squared test for categorical factors (Table 2). 

Multivariate logistic regressions were used to calculate the odds ratio (OR) and the 95% confidence interval (CI) of LBW for levels of SES (the highest SES was considered the reference) with adjustment for potential confounders (ethnicity, child’s gender, parity, maternal age at birth of the child) as shown in model 1. Thereafter, model 1 was adjusted for possible explanatory variables separately, to examine the association with LBW (third criterion) and also to examine the contribution of each possible explanatory variable to the explanation of social inequalities in LBW (fourth criterion). All mediators were added to model 1 to identify the total contribution of the mediating variables to the explanation of social inequalities in LBW. The change (decrease or increase) in the OR for parental SES with the higher risk of LBW was calculated with the formula 100 × ((OR_(model1)_ − OR_(adjusted model)_/(OR_(model1)_ − 1)) (Table 3) [65]. To examine the multivariate association between the mediators and LBW in children with PAE, logistic regression analysis was applied (Table 4). The Statistical Package of Social Sciences (SPSS) version 19.0 was used for all statistical analyses.

**Table 1:** Relation between independent and dependent variables, and potential mediators and confounders.

Total N = 2,159	High SESN = 829 (38.4%)	Middle SESN = 960 (44.5%)	Low SESN = 370 (17.1%)	*p* for Trend
*Pregnancy characteristics*				
Low birth weight (%)	5.8	8.9	11.1	0.004
Maternal age at birth of the child (years)	31.1 (4.5)	28.8 (4.8)	27.0 (5.0)	<0.001
Parity (% >0)	67.2	71.2	72.1	0.147
Gender (% male)	48.4	48.6	52.7	0.339
Ethnicity (%)				<0.001
German	87.8	91.3	78.5	
Slavic	2.2	3.6	7.1	
Turkish	0.4	1.2	6.3	
Others	9.6	4.0	8.2	
*Anthropometrics*				
Maternal height (cm)	168.4 (6.1)	167.1 (6.0)	165.6 (6.3)	<0.001
Paternal height (cm)	181.5 (7.1)	179.7 (7.1)	177.6 (7.1)	<0.001
Maternal BMI (% underweight)	2.2	2.1	3.9	0.151
Paternal BMI (% underweight)	0.3	0.4	0.4	0.920
Gestational gain in weight (%)				0.014
Low	17.3	17.0	19.3	
Medium	59.0	52.5	50.9	
High	23.7	30.5	29.8	
*Health-related behavior*				
Smoking during pregnancy (%)				<0.001
No	90.8	79.7	62.4	
Moderately	7.2	14.6	20.7	
Regularly	2.1	5.7	16.9	

Notes: BMI = body mass index. Data were missing for maternal age at birth of the child (N = 25), parity (N = 376), ethnicity (N = 12), maternal height (N = 25), paternal height (N = 239), gestational gain in weight (N = 149), maternal BMI (N = 37), paternal BMI (N = 271), and smoking during pregnancy (N = 9). Values are percentages for categorical factors, or means (with standard deviations) for continuous factors. *p*-values for trend are derived from chi-squared tests for trend (categorical factors) or from the linear trend test of the one-way analysis of variance.

## 3. Results

Table 1 shows the relation between the independent and dependent variables, and potential mediators and confounders. Of the 2,159 participants whose mothers consumed alcohol during pregnancy, 17.1% are from the low social class, 44.5% have a social middle-class background, and 38.4% are from the highest social class. The total prevalence of LBW was 8.1% and followed a social gradient, with the highest prevalence in the lowest SES group: prevalence rates of LBW are 5.8% in the high SES group, 8.9% in the middle SES group, and 11.1% in the low SES group (*p* for trend 0.004). Women with a higher SES were older when they gave birth to their child. Of the offspring with a high SES, 87.8% are German compared to 91.3% of the middle SES offspring and 78.5% of the low SES offspring. Parents from the highest social class are taller than parents from the middle and low social class (*p* < 0.001). The prevalence of low gestational weight gain was highest (19.3%) in the low social class with (*p* = 0.014). Concerning maternal health-related behavior during pregnancy, mothers from the low social class have the highest moderate and regular smoking rate during pregnancy (*p* for trend all ≤ 0.001). 

Table 2 presents the univariate associations between LBW and potential mediators. Women who gave birth to a child with LBW were shorter and had a higher prevalence of underweight (*p* < 0.001). The fathers of children with LBW were shorter than the fathers of children with a higher birth weight (*p* < 0.016). The prevalence of low gestational weight gain was 40.3% in women who gave birth to a child with LBW compared with a prevalence of 15.6% of low gestational weight gain in women who did not deliver a child with LBW (*p* < 0.001). 

**Table 2:** Relation between low birth weight and potential mediators.

Total N = 2,159	Low Birth Weight	No Low Birth Weight	*p* for
	N = 174 (8.1%)	N = 1,985 (91.9%)	Trend
*Anthropometrics*			
Maternal height (cm)	164.3 (5.9)	167.6 (6.1)	<0.001
Paternal height (cm)	178.8 (6.9)	180.3 (7.2)	0.016
Maternal BMI (% underweight)	6.6	2.1	<0.001
Paternal BMI (% underweight)	0.3	0.0	0.481
Gestational gain in weight (%)			<0.001
Low	40.3	15.6	
Medium	42.3	55.8	
High	17.4	28.6	
*Health-related behavior*			
Smoking during pregnancy (%)			<0.001
No	65.5	82.3	
Moderately	19.5	12.2	
Regularly	14.9	5.5	

Notes: BMI = body mass index. Data were missing for maternal height (N = 25), paternal height (N = 239), maternal BMI (N = 37), paternal BMI (N = 271), gestational gain in weight (N = 149), and smoking during pregnancy (N = 9). Values are percentages for categorical factors, or means (with standard deviations) for continuous factors. P-values for trend are derived from chi-squared tests for trend (categorical factors) or from the linear trend test of the one-way analysis of variance.

**Table 3:** Change in the odds ratio (OR) related to levels of socioeconomic status (SES) with low birth weight after separate adjustment for potential mediators.

	SES				
	High	Middle	Percentage Change ^c^	Low	Percentage Change ^b^
Model 1: maternal age at child’s birth, ethnicity, parity, child’s gender	1.00	2.04 (1.28, 3.24)		2.78 (1.59, 4.86)	
Model 1 + maternal height ^a^	1.00	1.91 (1.19, 3.06)	−12.5	2.18 (1.22, 3.90)	−33.7
Model 1 + paternal height	1.00	1.90 (1.16, 3.08)	−13.5	2.38 (1.28, 4.45)	−22.5
Model 1 + maternal BMI ^a^	1.00	2.14 (1.33, 3.42)	+9.6	2.64 (1.49, 4.68)	−7.9
Model 1 + paternal BMI	1.00	1.90 (1.17, 3.08)	−13.5	2.62 (1.41, 4.87)	−9.0
Model 1 + gestational gain in weight ^a^	1.00	2.28 (1.37, 3.78)	+23.1	2.86 (1.54, 5.29)	+4.5
Model 1 + smoking during pregnancy ^a^	1.00	1.86 (1.17, 2.98)	−17.3	2.22 (1.25, 3.96)	−31.5
Model 1 + maternal height, maternal BMI, smoking during pregnancy	1.00	1.79 (1.11, 2.89)	−24.4	1.71 (0.94, 3.13)	−60.1

Notes: BMI = body mass index. Values are ORs with 95% confidence intervals. ^a^ Significant relation to low birth weight (*p* < 0.05). ^b^ Change in OR relates to the change of the OR relative to model 1 for middle and low SES after separate adjustment for potential mediators 100 × ((OR_(model 1)_ − OR_(adjusted model)_)/(1 − OR_(model 1)_)).

**Table 4:** Logistic regression model fitted on low birth weight.

	OR (95% CI)
*SES*	
High (reference)	1.00
Middle	1.79 (1.11, 2.89)
Low	1.71 (0.94, 3.13)
Maternal age at birth of the child (years)	1.01 (0.97, 1.06)
*Parity*	
0 (reference)	1.00
>0	0.49 (0.32, 0.75)
*Gender*	
Male (reference)	1.00
Female	1.60 (1.08, 2.35)
*Ethnicity*	
German (reference)	1.00
Slavic	1.24 (0.55, 2.80)
Turkish	1.40 (0.46, 4.30)
Others	1.43 (0.72, 2.84)
Maternal height (cm)	0.91 (0.88, 0.95)
*Maternal BMI*	
Not underweight (reference)	1.00
Underweight	2.53 (1.04, 6.13)
*Smoking during pregnancy*	
No smoking (reference)	1.00
Moderate smoking	1.55 (0.92, 2.63)
Regular smoking	2.52 (1.33, 4.75)
Constant B (SE)	11.582 (2.915)
N	1727
Nagelkerke’s R²	0.106

Note: BMI = body mass index. Values are odds ratios (ORs) with 95% confidence intervals.

Table 3 shows socioeconomic differences in LBW and the contribution of each potential explanatory factor to the explanation of social inequalities in LBW in children with PAE. The decrease in the ORs for SES indicates the explanatory power of the variables to the socioeconomic differences in LBW. When comparing LBW in the middle and low SES group with the high SES group, the adjusted OR for the low SES group was 2.78 (95% CI 1.59, 4.86) and the adjusted OR for the middle SES group was 2.04 (1.28, 3.24). The separate adjustment for anthropometric and health-related factors shows that maternal height, maternal BMI and smoking during pregnancy, mediated the relationship between SES and LBW. After adjustment for confounders, paternal height and paternal BMI did not remain significantly related to LBW. Gestational gain in weight remained significantly related to LBW after adjustment for confounders, but did not result in a decrease of the ORs. Therefore, this variable could not be accepted as a mediator in the socioeconomic differences of LBW. The mediating effect of maternal height was 12.5% in the middle SES group and 33.7% in the low SES group. Maternal BMI caused a 7.9% decrease in the ORs in the low SES group. The mediating effect of smoking during pregnancy was 27.9% in the middle SES group and 31.5% in the low SES group. When all mediating variables, maternal height, maternal BMI and smoking during pregnancy, were included in the model, the OR decreased to 1.79 (95% CI 1.11, 2.89) in the middle SES group and to 1.71 (0.94, 3.13) in the low SES group, implying a decrease of 24.4% and 60.1%, respectively.

Table 4 presents the model including all mediating variables from Table 3 (model 1 + maternal height, maternal BMI, smoking during pregnancy) to demonstrate the associations between LBW in children with PAE and the mediating variables. The risk of LBW decreased with increasing maternal height (OR 0.91, 95% CI 0.88, 0.95). The risk of LBW was greater in women with underweight (OR 2.53, 95% CI 1.04, 6.13). Also, the risk of LBW increased in offspring exposed to alcohol whose mother also smoked regularly (OR 2.52, 95% CI 1.33, 4.75).

## 4. Discussion

### 4.1. Main Findings

In this study, the risk of LBW was increased in the offspring with PAE from the low and middle social class compared with those from the high social class. Maternal height and smoking during pregnancy mediated the relationship between the SES and LBW in children with PAE. Maternal height explained 12.5% to 33.7% of the socioeconomic differences in LBW in children with PAE. Maternal BMI explained 7.9% of the socioeconomic differences in LBW between the low and high SES group in children with PAE. Smoking during pregnancy explained 17.3% to 31.5% of the socioeconomic differences in LBW in children with PAE. Both factors together contributed 24.4% to 60.1% to the explanation of socioeconomic differences in LBW in children with PAE. 

### 4.2. Strengths and Limitations

The study is based on data from a very large and representative national sample. Studies on the reliability of alcohol measurements have demonstrated that drinking during pregnancy is often underreported [66,67,68,69]. However, the present study included only subjects with moderate to regular PAE and included a relatively large sample of 2,159 individuals. 

The sample in this study could be confounded by various factors. For example, if reports on alcohol intake during pregnancy are related to levels of SES, the social distribution of alcohol-exposed individuals could be biased in unknown ways. Also, if reports on alcohol intake during pregnancy are related to the health status of the child, or to the health status and/or to the health behavior of the mother, or to all of these aspects, the estimates could be biased in unknown ways.

In the present study, maternal health-related behavior during pregnancy was restricted to smoking during pregnancy. Additional health-related behaviors (such as caffeine intake during pregnancy and dietary factors) might be relevant in the explanation of why children exposed to similar levels of alcohol before birth from the lower social class are at higher risk for LBW compared with offspring from the upper class. Therefore, additional studies are needed to investigate the role of a wider range of health-related behaviors during pregnancy in the explanation of social inequalities in LBW in children with PAE.

Because smoking during pregnancy was self-reported, underreporting might have occurred [70,71,72,73]. Therefore, the association between smoking during pregnancy and LBW might be underestimated and the “real” effect of smoking during pregnancy on LBW might be even stronger. If reports on smoking during pregnancy are associated with levels of SES, the results could be biased in unknown ways.

The multiple regression model only explained 10.6% of the variance in LBW, even though it was controlled for a large number of confounders. 

### 4.3. Discussion of the Main Findings

The risk of LBW in the offspring of self-reported moderate to regular drinkers during pregnancy was greater in those with a low and middle SES. This result is in line with others who reported social inequalities in the effects of maternal alcohol intake during pregnancy [18,19,20,21,22,23,24,25]. This finding is also in agreement with another study showing decreased birth weight in alcohol-exposed subjects from the lower social class compared with the upper social class [20]. 

The present study has shown that the single effect of smoking during pregnancy explains a substantial amount (17.3% to 31.5%) of the social gradient in LBW in children with PAE. The ingredients of tobacco smoke (e.g., carbon monoxide and nicotine) cause a decrease in birth weight as these factors are responsible for a reduction in blood flow/oxygen content, and nicotine exposure results in contraction of the placental vessels [74,75,76,77]. The considerable explanatory power ascribed to socioeconomic differences in LBW in children with PAE, that can be attributed to smoking during pregnancy, might be due to the greater toxic effects of smoking in subjects with PAE. Alcohol and tobacco smoke together are reported to have a greater toxic effect on birth weight, as the effect of alcohol is exacerbated by tobacco smoke [18,19,43,44,45,46]. Therefore, the role of smoking during pregnancy in the explanation of socioeconomic differences in LBW likely has considerable relevance and, thereby, contributes to our understanding of why children with PAE from the lower social class have a higher risk of LBW.

The present study shows that the single effect of maternal height explains a considerable amount (12.5% to 33.7%) of the social gradient in LBW in children with PAE. In agreement with others [37,38,78,79,80,81], the present study shows that taller women have a lower risk of giving birth to a child with LBW. The large explanatory power to socioeconomic differences in LBW in children with PAE that can be attributed to maternal height might be due to lower levels of BAC in taller women, resulting in lower levels of BAC in the fetus [23]. Thus, maternal height is likely to modify the effect of alcohol on the embryo/fetus; future research should investigate potential effect modifications of PAE by maternal height. The role of maternal height in the explanation of social inequalities in LBW appears to be relevant and helps elucidate why children with PAE from the lower social class are at higher risk of LBW.

Maternal BMI explains 7.9% of the socioeconomic difference in LBW between the high and the low SES group in children with PAE; the finding of an increased risk of LBW in women with underweight concurs with other studies [37,82] and with a recent meta-analysis [83]. Underweight women might have a lack of growth-stimulating nutrients, which are responsible for the reduced birth weight of the embryo/fetus. Maternal BMI contributed to the explanation of socioeconomic differences in LBW in the low SES group only. Of the women in the present study, the prevalence of underweight was higher in the low social class than in the middle/high social class, but almost similar in the middle and high social class. Therefore, the explanation of the social gradient in LBW by maternal BMI is limited to the low SES group only. 

Maternal anthropometrics, in terms of maternal height and BMI, as well as maternal health-related behavior, in terms of smoking during pregnancy, contributed to the explanation of social inequalities in LBW among children with PAE. A considerable amount (24.4% to 60.1%) of the socioeconomic differences in LBW in children with PAE was explained by health-related behavioral and anthropometric factors together. Both factors seem to be of importance for the explanation of social inequalities in LBW in children with PAE. 

## 5. Conclusions

This study has shown that LBW in children with PAE is subject to social inequalities. Maternal health-related behavior during pregnancy and maternal anthropometrics make a considerable contribution to our understanding of socioeconomic differences in LBW in children with moderate to regular PAE. LBW acts as a catalyst for diseases throughout the life course. The development of FASD in alcohol-exposed children is related to LBW and this study shows that smoking during pregnancy, lower maternal height, and being underweight trigger the risk of LBW. Both anthropometric and health-related behavioral factors might mediate the association between SES and alcohol-related disorders. To reduce social inequalities in the health of children with PAE at an early stage, it is important to inform women with a lower SES about the adverse effects of tobacco smoke and being underweight on the embryo/fetus. However, prevention campaigns related to the adverse effects of alcohol and tobacco smoke during pregnancy should be designed to reach all women from all social classes.
